# Influence of Feature Encoding and Choice of Classifier on Disease Risk Prediction in Genome-Wide Association Studies

**DOI:** 10.1371/journal.pone.0135832

**Published:** 2015-08-18

**Authors:** Florian Mittag, Michael Römer, Andreas Zell

**Affiliations:** Cognitive Systems Group, University of Tübingen, Tübingen, Germany; CSIR-Institute of Microbial Technology, INDIA

## Abstract

Various attempts have been made to predict the individual disease risk based on genotype data from genome-wide association studies (GWAS). However, most studies only investigated one or two classification algorithms and feature encoding schemes. In this study, we applied seven different classification algorithms on GWAS case-control data sets for seven different diseases to create models for disease risk prediction. Further, we used three different encoding schemes for the genotypes of single nucleotide polymorphisms (SNPs) and investigated their influence on the predictive performance of these models. Our study suggests that an additive encoding of the SNP data should be the preferred encoding scheme, as it proved to yield the best predictive performances for all algorithms and data sets. Furthermore, our results showed that the differences between most state-of-the-art classification algorithms are not statistically significant. Consequently, we recommend to prefer algorithms with simple models like the linear support vector machine (SVM) as they allow for better subsequent interpretation without significant loss of accuracy.

## Introduction

State-of-the-art genotyping platforms are able to measure hundreds of thousands or even millions of single nucleotide polymorphisms (SNPs) at once, giving rise to a steadily increasing amount of data being generated [1]. They also led to an increasing number of Genome-wide Association Studies (GWAS) that unveiled novel susceptibility variants or pointed towards putative loci in different diseases [2, 3]. Based on this information, researchers continue to gain knowledge about cellular and genetic mechanisms [4–8]. Apart from providing a better understanding of diseases, this also enabled geneticists to develop models for genetic risk profiling [9–12]. If a genetic predisposition for certain diseases can be identified at a young age, life-style changes and therapies can be implemented at an early stage and delay or even prevent the onset of some diseases [13, 14]. In some cases, the therapy itself can be tailored individually to the patient’s genetic traits, e.g., by fine-tuning the dosage of medication to maximize its effect while minimizing side-effects [15–17].

Over time, more and more sophisticated analysis methods have been developed or adapted to detect markers associated with certain phenotypes in these very large data sets [18, 19]. Besides the standard single marker statistics, modern machine learning algorithms have been employed in various studies to find risk factors or to build models for predicting the disease risk [20–22]. Early studies first gathered already known genetic markers and counted the number of causal variants in a sample, calculating a score that represented the disease risk [23, 24]. State-of-the-art classification algorithms can be viewed as an automated enhancement of this strategy, but the specifics vary between different algorithms [25]. They weigh and select the features with the highest information content and build models that act as a decision function to distinguish between two or more classes as best as possible. Of course, their application is not limited to genetic data, but data of all kinds and fields and numerous studies have investigated these algorithms in different scenarios [26–28]. Nonetheless, the performance of these classification algorithms depends on the type of data they are applied to, so there is no “best” algorithm for everything [29] and various algorithms should be evaluated to identify the ones most fit for a specific problem type.

Trying to predict the individual disease risk based only on SNP genotype information [9, 30] was the next logical step. These SNP data can also be represented using different encodings that may influence the training of good classifiers. A study from [20] applying support vector machines (SVMs) on type 1 diabetes data sets found no difference between the additive and the recessive/dominant encoding scheme. However, Miller et al. showed that a recessive/dominant encoding of SNPs can be superior to additive schemes when capturing epistatic effects [31]. Thus, the influence that the encoding scheme or even the specific algorithm may have on the performance of those models can hopefully provide further insight into the underlying genetic structure of the investigated diseases. While there have been studies that compared the predictive performance of multiple algorithms [32], they did so without the use of statistical methods designed for multiple comparisons. To our knowledge, the suitability of different encodings, especially in conjunction with various classification algorithms, has not been assessed systematically before.

In this study, we focused on seven GWAS case-control data sets from the Wellcome Trust Case-Control Consortium (WTCCC) that, except for binary phenotype, gender and sample ID, contain only SNP allele information [33]. Similar to the selection schemes in classical genome-wide association studies [34, 35], we selected those SNPs as input features that reached a certain predefined genome-wide association threshold. We systematically evaluated three different feature encodings and seven machine learning algorithms with regard to their predictive performance. Using the best of these three encodings we then compared the performance of the algorithms via the Friedman test [36], a non-parametric statistical test for multiple comparisons, and Shaffer’s static post-hoc test [37] for the subsequent pair-wise comparison.

## Materials and Methods

### Data Sets

The Wellcome Trust Case Control Consortium (WTCCC) offers GWAS data sets on various diseases with ca. 2,000 samples and two control cohorts with ca. 1,500 samples each [33]. In our study, we included the studies for bipolar disorder (BD), Crohn’s disease (CD), coronary heart disease (CAD), hypertension (HT), rheumatoid arthritis (RA), type 1 and type 2 diabetes (T1D and T2D) and the 1958 British Birth Cohort (58C) as controls. The genotyping of those data sets was conducted using the Affymetrix 500K chip.

We also applied stringent quality control measures to reduce the risk of artifacts. First, we removed all SNPs that were on a list of bad markers provided by the WTCCC. Additionally, SNP rs41388745 was removed because it was ambiguously mapped to two different chromosomes. Since this study focused on the impact of different feature encodings and their implication on the assumed genetic risk model, we did not include the X-chromosome. Although novel ways to deal with its special characteristics have been proposed [38], a standardized way of evaluating those markers has not been established [39].

Each disease and the control data set was then analyzed separately using PLINK [40]. We excluded SNPs with a minor allele frequency (MAF) less than 5%, a genotyping rate of less than 5% or a significant deviation from the Hardy-Weinberg equilibrium (*p* < 0.001). This process was then repeated for each combined data set consisting of one disease and the control data set.

### Data Encoding

The SNP data can be represented as nominal features, e.g., *AA*, *AG* or *GG*, or numerical, e.g., 0, 1 and 2. While some classification algorithms can work with nominal features, like the Decision Tree or the Random Forest, almost all can work on numerical ones and some can work *only* on those, like the Support Vector Machine or the Multilayer Perceptron. This makes it necessary to encode the SNPs as numerical features. There are different ways to encode SNPs and each encoding may represent different biological assumptions. The encoding can also affect the ability of machine learning algorithms to learn a model (see Machine Learning Algorithms).

In the *additive* model, each genotype is encoded as a single numeric feature that reflects the number of minor alleles. Homozygous major, heterozygous and homozygous minor are encoded as 0, 1 and 2, respectively. This results in a minimal number of generated features while preserving all information. On the other hand, non-additive effects like heterozygosity leading to a higher disease risk than homozygosity cannot be modeled by non-linear classifiers.

In the *recessive/dominant* model, each genotype is encoded as two binary features, one for each possible allele. A feature is set to 0 if the corresponding allele is not present and set to 1 if it is present at least once. This doubles the number of features, which requires more memory and can increase the computation time. However, this encoding can be superior to the additive encoding when modelling multi-locus interactions [31].

Another binary way to encode the genotype information is to create three features for each SNP, one for each genotype, which has also proven to be useful for detecting gene-gene interactions [19]. In this encoding, each feature represents whether its corresponding genotype is present or not, which means that exactly one of the three features is 1 and the other two are 0. While this scheme is also called *one-hot encoding*, we denote this scheme as *genotypic* to reflect the implications on the risk model. Since each genotype is represented by a separate feature, classifiers can build more fine grained models. The downside is an even higher memory consumption due to the redundancy of this feature representation.

In this study, we focused on these three encoding schemes. An example is illustrated in Table 1.

**Table 1:** Illustration of the three different encoding schemes for SNP data.

SNP_i_	Add count	Rec		Gen		
		A	B	AA	AB	BB
AA	0	1	0	1	0	0
AB	1	1	1	0	1	0
BB	2	0	1	0	0	1

We investigated three different encoding schemes for SNP data, here with two alleles A (major) and B (minor). The *additive* encoding (Add) represents each genotype through the minor allele count. The *recessive/dominant* (Rec) encoding encodes the presence of at least one allele for each of the two. The *genotypic* (Gen) encoding consists of three features, one for each possible genotype.

### Machine Learning Algorithms

We used the Weka data mining framework [41] version 3.6.4 to perform all machine learning tasks, because it contains implementations of many state-of-the-art machine learning algorithms. It was also responsible for all data management necessary for the pre-processing of the data sets and collecting the results of the different classifiers. This ensured that each machine learning algorithm processed the same data, i.e. same randomization, filtering and normalization. Weka and its algorithms are implemented in Java, giving the additional benefit of platform independence.

The algorithms compared in this study were *k*-nearest neighbor (kNN), decision tree (DT), random forest (RF), multilayer perceptron (MLP), learning vector quantization (LVQ) and support vector machine (SVM). In addition to the implementations included in Weka we extended the MLP to offer the option to set the number of hidden nodes to the square root of the number of features. We also wrote our own Weka wrapper for the LibSVM implementation [42] of the SVM because the available ones did not report the AUC correctly. The LVQ implementation was provided through a plug-in (see Table 2).

**Table 2:** Algorithm implementations.

Algorithm	Weka class
Decision Tree	weka.classifiers.trees.J48
Random Forest	weka.classifiers.trees.RandomForest
SVM (linear)	own LibSVM wrapper
SVM (RBF)	own LibSVM wrapper
kNN	weka.classifiers.lazy.IBk
Multilayer Perceptron	weka.classifiers.functions (modified^1^)
LVQ	weka.classifiers.neural.lvq.MultipassLvq^2^

^1^: Added the option to use $\sqrt{\#SNPs}$ hidden nodes instead of fixed number

^2^: Plug-in from http://wekaclassalgos.sourceforge.net/

The kNN algorithm is one of the simplest and provides a baseline for the others, LVQ is related to KNN, but has some advantages regarding model interpretation. RF, MLP and SVM are popular state-of-the-art classification algorithms that work in fundamentally different ways. We also included the DT because it is the precursor to the RF. In addition to the standard linear SVM we also included a non-linear one using the radial basis function (RBF) kernel [43].

Classifiers can be linear or non-linear. A classifier is linear when it uses a linear combination of the features for its decision. The only algorithm tested in this study that falls into this category was the linear SVM. The advantage of linear classifiers is that the trained models are usually easier to interpret, because each feature has a corresponding weight. The obvious disadvantage of a linear classifier is that it cannot learn models that are non-linear in the feature space. This problem can be partially addressed by applying a transformation of the input features into the feature space, as it is the case with the recessive/dominant or the genotypic encoding.

Non-linear classifiers are capable of learning more complex models, which the linear classifiers can not. On the other hand, the subsequent interpretation of these models can be very complicated, if at all possible, depending on the used algorithm.

### Classifier Training and Evaluation

Each case-control data set was comprised of one of the seven disease data sets (approx. 2,000 cases each) and the 1958 British Birth Cohort data set (1,500 controls). We chose the area under the ROC curve (AUC) as the performance measure due to the imbalanced class ratio. The predictive performance was measured via a 5 × 2 cross-validation (a 2-fold cross-validation with five repetitions) and the results were averaged. The averaging was necessary because the statistical test applied afterwards requires independent measurements, which is not the case for random subsamples of the same data set. It should also be noted that the random splits of each case-control data set were kept the same during all subsequent processing steps to ensure comparability.

During the 5 × 2 cross-validation we computed five different single marker statistics on the training set of each fold, which mirror the genome-wide association tests of PLINK when using the --model option: Allelic, recessive, dominant and genotypic test and the Cochran-Armitage trend test. In each round of this outer cross-validation, the respective pair of training and test set was filtered with six different *p*-value thresholds. Only those SNPs with a *p*-value reaching the threshold of 10^−3^, 10^−4^, 10^−5^, 10^−6^, 10^−7^ and 10^−8^ for at least one of the genome-wide association models were retained.

After that, the remaining SNPs were transformed into features according to the encoding scheme of the setting and then standardized to have zero mean and unit variance (*μ* = 0, *σ* = 1). Finally, all seven classification algorithms were evaluated on each of these filtered training-test set pairs. Additionally, to ensure that each machine learning algorithm performed at its best, an exhaustive parameter optimization was performed using a 5-fold inner cross-validation for each parameter combination (see Table 3). The parameter combination with the highest AUC was then used for the outer cross-validation. In total, this yielded 7 diseases × 6 thresholds × 3 encodings × 7 algorithms = 882 different experimental settings. Each of these settings was evaluated using a 5 × 2 outer cross-validation, where for each fold the parameters were selected using a 5-fold inner cross-validation.

**Table 3:** Parameter optimization values.

Algorithm	Parameter	Values
Decision Tree	Pruning at confidence c*	0.01, 0.03, 0.05, 0.1, 0.2, 0.3, 0.4, Reduced error pruning, No pruning
Random Forest	No. of trees	10, 30, 100, 300, 1000
SVM (linear)	C	2^−5^, 2^−3^, …, 2^11^
SVM (RBF)	C	2^−5^, 2^−3^, …, 2^11^
	*γ*	2^−15^, 2^−13^, …, 2^3^
kNN	No. of nearest neighbors Weighting	1, 3, 10, 30, 100 constant / inverse of distance
Multilayer Perceptron	Learning rate	0.01, 0.03, 0.1, 0.3
	Learning rate decay	yes / no
	No. of hidden units	2, 4, 8, 16, 32, $\sqrt{\#SNPs}$
LVQ	none	multipass; automatic selection

*: Pruning at confidence level *c*, reduced error pruning and no pruning are mutually exclusive

Parameter optimization was conducted for for each fold of the outer cross-validation. Each combination of the parameters was evaluated in a 5-fold inner cross-validation. Default values were used for all parameters not listed here.

### Statistical Evaluation

To determine whether some encodings or classification algorithms are significantly better than the others, we employed the *Friedman test* (sometimes called Friedman’s ANOVA). The Friedman test is the non-parametric equivalent of the repeated measures ANOVA and was used here because it does not make assumptions about the distribution of values. It tests against the null hypothesis that there are no significant differences between multiple treatments over a number of experiments.

It works by ranking the classification performance of *k* different treatments for each experiment *i* from best (rank 1) to worst (rank *k*). The ranks are denoted as $r_{i}^{j}$ with 1 ≤ *j* ≤ *k* and 1 ≤ *i* ≤ *n*. For each treatment *j* the average rank *R*_*j*_ is calculated as $R_{j}=\frac{1}{n}\sum_{i}\;r_{i}^{j}$. The Friedman test statistic *F* is then obtained via the formula

$$F=\frac{12n}{k(k+1)}\;[\sum_{j}R_{j}^{2}-\frac{k{\left(k+1\right)}^{2}}{4}]$$ (1)

and is approximately *χ* ^2^ distributed with *k* − 1 degrees of freedom, if *n* and *k* large enough (*n* > 10 and *k* > 5). However, we used a derived statistic, proposed by [44],

$$F_{ID}=\frac{\left(n-1\right)F^{2}}{n\left(k-1\right)-F^{2}}$$ (2)

that is less conservative and more accurate than the former [45]. It is distributed according to the F distribution with *k* − 1 and (*k* − 1)(*n* − 1) degrees of freedom and *p*-values were obtained by comparing *F*_*ID*_ against this distribution.

Our study followed a repeated measures design because for each *p*-value threshold each data set was randomized for cross-validation once and then evaluated for each encoding and algorithm. Due to the 5 × 2 cross-validation, there were 10 values for each combination of treatment, data set and threshold. It was tempting to treat each of these 10 values as a separate learning problem, as it would increase the number of samples and thus the power of the subsequent statistical test. However, while the Friedman test requires the measures to be matched between the algorithms, the samples must be independent from each other. This was clearly not the case, since each of the 10 training sets was sampled from the same underlying data set. Thus, the results of a 5 × 2 cross-validation were averaged and used as a single measurement [46].

A potentially problematic aspect regarding the independence of samples was filtering the data set using different thresholds. For each data set and each algorithm, six different learning problems are generated by including only SNPs reaching a certain *p*-value threshold, creating nested subsets where each set contains all SNPs of the sets resulting from a smaller *p*-value threshold. We recorded the number of SNPs used for each data set and threshold to be able to investigate whether a potentially concerning dependence of features was present. To decrease the risk of false positive findings, we choose a stringent significance level *α* = 0.001.

As with the standard ANOVA, the Friedman test is an omnibus test and can only detect whether there are differences in the overall comparison. If significant differences are found, pair-wise comparisons of the average rankings have to be conducted to find out which of the treatments differ among each other. The test statistic for testing the hypothesis that treatments *i* and *j* are not different can be calculated by

$$z=\left(R_{i}-R_{j}\right)/\sqrt{\frac{k(k+1)}{6n}}$$ (3)

and the *p*-value can be obtained by normal approximation. However, those *p*-values have to be corrected for multiple testing because *m* = *k*(*k* − 1)/2 pair-wise comparisons are performed this way.

Various post-hoc methods exist for calculating the adjusted *p*-values. The Nemenyi test simply multiplies the uncorrected *p*-values with the number of pair-wise comparisons [47]. We used Shaffer’s static method in our study [37] since it is more powerful than the Nemenyi test [48]. The basic principle of Shaffer’s static method is that when testing *k* treatments for equality or inequality, not all combinations are possible. For example, when the hypothesis that two out of three treatments are different is false, at least one of the other two hypotheses must be false, too. So instead of multiplying each *p*-value with the number of pair-wise comparisons *m*, Shaffer’s static method follows a step-down approach: First, the *m* pair-wise hypotheses that there is no difference between two specific treatments are sorted by their corresponding *p*-values from best (smallest) to worst (highest). Then, starting at the best *p*-value, each one is multiplied by the number of hypotheses that can be true given that all previous hypotheses have been rejected. This method is called static because the number of possible true hypotheses is independent of the actual *p*-values and must be calculated only once. To decrease the risk of false positive findings, we choose a stringent significance level *α* = 0.001 in all tests.

## Results

The presentation of the results in this section is divided into one part for the comparison of different feature encodings and one for the comparison of classification algorithms. Statistics about the numbers of SNPs that reached the different *p*-value thresholds for the seven disease data sets are listed in Table 4. The raw AUC values representing the predictive performances of each learned model before ranking them are contained in S1 and S2 Tables.

**Table 4:** Average number of SNPs reaching the specified *p*-value threshold per data set.

*p*-value threshold	BD	CAD	CD	HT	RA	T1D	T2D
*p* < 10^−8^	1.0	3.4	3.2	1.0	60.5	102.8	1.4
*p* < 10^−7^	1.3	4.3	4.9	3.5	73.6	123.5	2.2
*p* < 10^−6^	3.8	8.0	11.3	6.4	89.5	153.5	5.4
*p* < 10^−5^	22.9	23.1	40.9	22.2	126.0	212.7	20.2
*p* < 10^−4^	149.7	129.0	221.2	131.5	280.8	391.9	132.5
*p* < 10^−3^	1238.3	1047.9	1560.0	1081.5	1332.0	1398.3	1115.4

Average number of SNPs reaching the specified *p*-value thresholds for at least one of the tests for genome-wide association. Numbers are averaged over all 10 results of the 5 × 2 cross-validations and rounded to one decimal place.

### Encodings

Comparison of the three different encodings additive (Add), recessive/dominant (Rec) and genotypic (Gen) reveal a clear advantage of the additive encoding in terms of predictive performance. The null hypothesis that there is no difference between the different encodings can be rejected with *p* < 10^−15^ (see Table 5) after applying the Friedman Test on all data sets for all classification algorithms (*k* = 3, *n* = 42). The subsequent pair-wise comparison of the encodings shows that the additive encoding is better than the other two, but there is no statistically significant difference between the recessive/dominant and the genotypic encoding (see Table 6).

**Table 5:** Average ranks and *p*-values of the Friedman test for the three encoding schemes.

	Add	Rec	Gen	*p*-value
all	1.53	2.12	2.35	< 10^−15^
Lin	1.21	2.20	2.58	4.819 ⋅ 10^−13^
RBF	1.25	2.50	2.25	5.689 ⋅ 10^−11^
MLP	1.56	1.85	2.60	9.957 ⋅ 10^−7^
DT	1.88	2.00	2.12	0.557
RF	1.63	2.13	2.24	0.01058
KNN	1.65	1.82	2.52	5.51 ⋅ 10^−5^
LVQ	1.55	2.31	2.14	0.0007697
BD	1.76	2.18	2.06	0.1447
CAD	1.67	2.17	2.17	0.02823
CD	1.38	2.10	2.52	6.029 ⋅ 10^−8^
HT	1.74	2.29	1.98	0.04019
RA	1.38	2.00	2.62	2.485 ⋅ 10^−9^
T1D	1.10	2.07	2.83	< 10^−15^
T2D	1.71	2.01	2.27	0.03522

Average ranks and *p*-values of the Friedman test for the three encoding schemes additive (Add), recessive/dominant (Rec) and genotypic (Gen). The average ranks are computed over all experimental settings or only those for a certain classifier or a certain disease data set. (Small values are better.)

**Table 6:** Rank differences and *p*-values for pair-wise comparison of encodings.

Hypothesis	Rank diff	*p*-value
Add vs Gen	0.82	< 10^−15^
Add vs Rec	0.58	1.765 ⋅ 10^−12^
Rec vs Gen	0.23	0.004434

For each pair-wise comparison of encodings, the rank difference was calculated as the difference of the average ranks over all data sets and algorithms. The first encoding of each hypothesis is the better one (lower rank). *p*-values were corrected using Shaffer’s static method.

**Fig 1:**
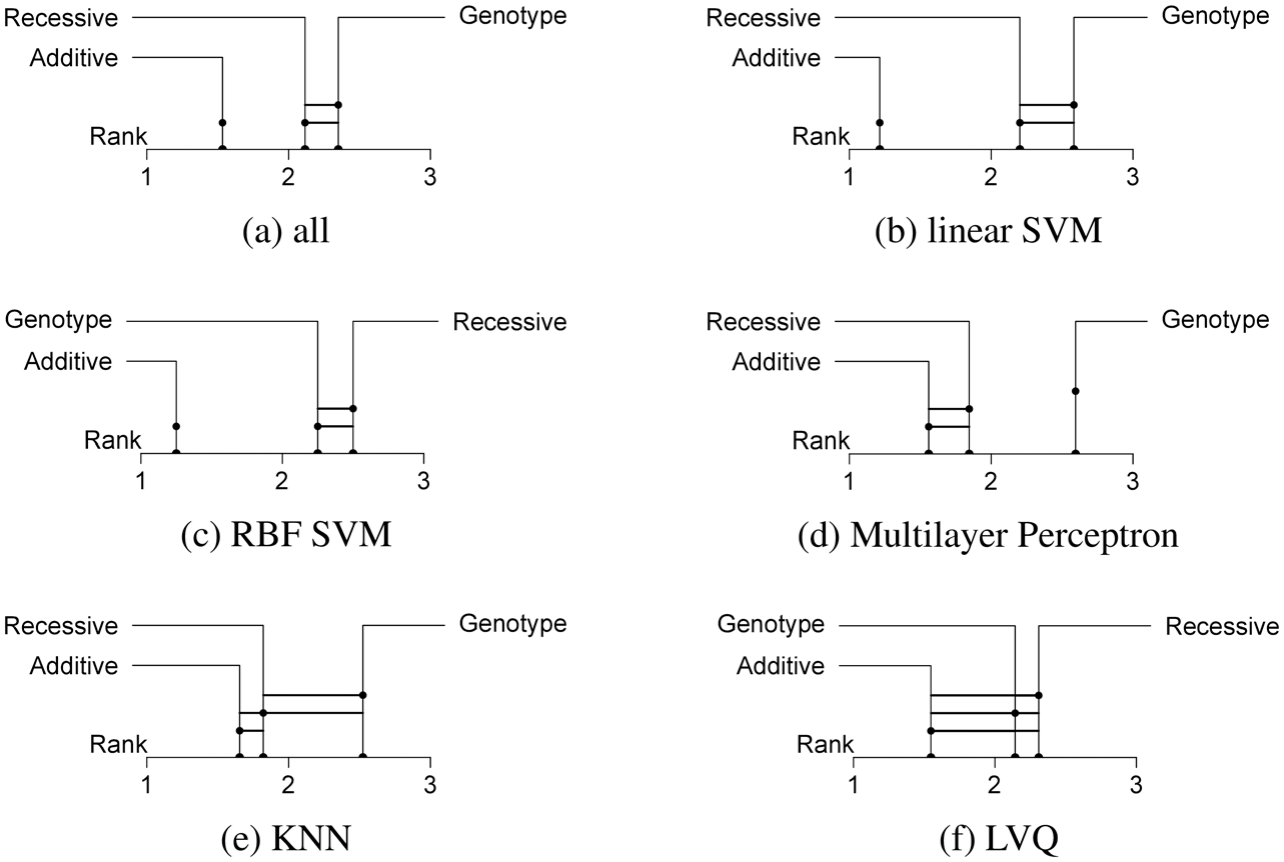
Comparison of encodings per classifier. The three encodings compared by their rank distance over all data sets and classifiers (a) and grouped by classifier. A connecting line between encodings means that the null hypothesis of them being significantly different could not be rejected. Only algorithms for which the Friedman test rejected the null hypothesis are shown. (*α* = 0.001.)

The comparison of encodings grouped by algorithms (*k* = 3, *n* = 6) also showed that the additive encoding always achieved the best average rank (see Fig 1). By far the largest differences could be observed for both SVMs, for the standard linear SVM (Fig 1b) as well as for the SVM with RBF kernel (Fig 1c) with *p*-values of 4.819 ⋅ 10^−13^ and 5.689 ⋅ 10^−11^, respectively. In both cases, the additive encoding achieved the best rank throughout all classifiers (1.21 and 1.25) and the next best encoding had a rank of at least 2. Also, the difference between recessive/dominant and genotypic encoding was not large enough to be statistically significant in both cases.

Next largest differences were found for the Multilayer Perceptron (Fig 1d, *p* = 9.957 ⋅ 10^−7^) and the k-nearest neighbor (Fig 1e, *p* = 5.51 ⋅ 10^−5^). Here the post-hoc test could not find significant differences between the additive and the recessive/dominant encoding, but between the additive and the genotypic encoding. It should also be noted that even though the Learning Vector Quantization came out significant (*p* = 7.697 ⋅ 10^−4^), Shaffer’s static method could not detect significant differences between any of the encodings (Fig 1f), which can be attributed to both tests differing in their statistical power. The null hypothesis could not be rejected for the Decision Tree (*p* = 0.557) and the Random Forest (*p* = 0.01058) algorithms.

**Fig 2:**
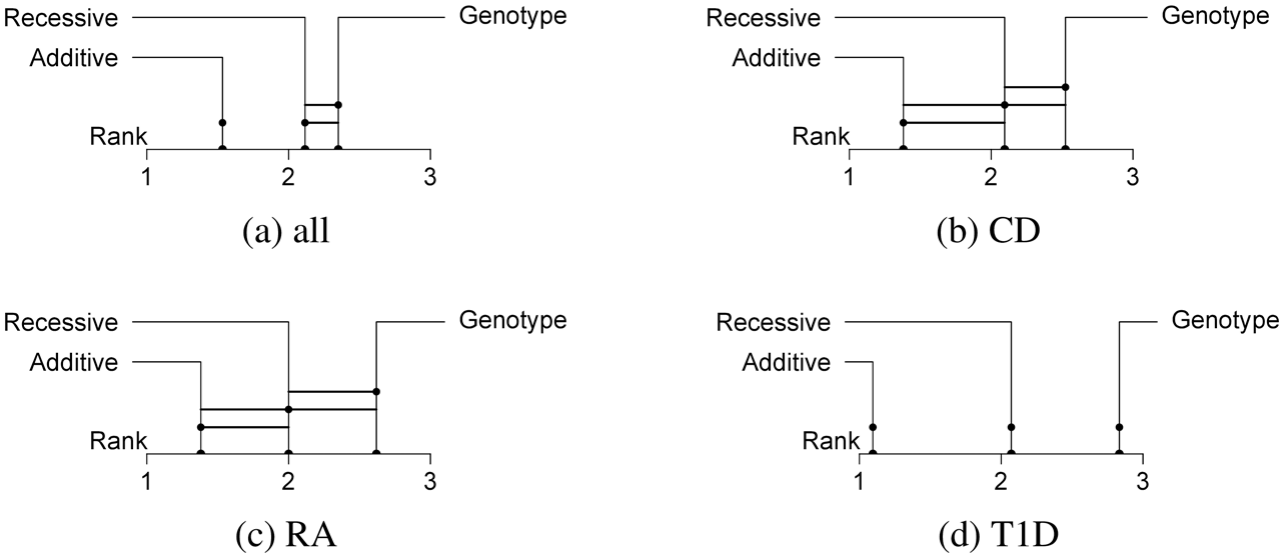
Comparison of encodings per disease data set. The three encodings compared by their rank distance over all data sets and classifiers (a) and grouped by disease data set. A connecting line between encodings means that the null hypothesis of them being significantly different could not be rejected. Only data sets for which the Friedman test rejected the null hypothesis are shown. (*α* = 0.001.)

The additive encoding also achieved the best average rank when looked at for each of the diseases separately (see Fig 2). For most diseases the Friedman test showed no significance (*k* = 3, *n* = 6). The three disease data sets with significant results were T1D (*p* < 10^−15^), RA (*p* = 2.485 ⋅ 10^−9^) and CD (*p* = 6.029 ⋅ 10^−8^). Especially the T1D data set, which had the best predictions by far, showed the biggest difference between all three encodings. It was the only single disease data set for which all pair-wise rank differences were significant. Also, the encodings were ranked in the same order in so many cases that the average ranks were very close to the discrete ranks (1.10, 2.07 and 2.83) The other two data sets for which the Friedman test rejected the null hypothesis only reported a significant difference between the additive and the genotypic encoding. Apart from that, two observations could be made for the disease data sets: Firstly, when differences were detected, it was always the additive encoding that outperformed the genotypic encoding. Secondly, recessive/dominant and genotypic encoding only differed significantly for the T1D data set.

We also compared the average and the maximum AUCs of the three encodings for each disease data set (see Table 7). In all cases the additive encoding had the highest average and the highest maximum AUC, while the recessive/dominant and genotypic encoding showed no consistent ordering.

**Table 7:** Maximum and average AUCs for different encodings grouped by data set.

AUC		BD	CAD	CD	HT	RA	T1D	T2D
Max	Add	0.5834	0.5843	0.6178	0.5617	0.7276	0.8682	0.5861
	Rec	0.5752	0.5753	0.6158	0.5517	0.7191	0.8558	0.5837
	Gen	0.5752	0.5771	0.6080	0.5559	0.7167	0.8521	0.5773
Avg	Add	0.5383	0.5571	0.5793	0.5226	0.6736	0.8031	0.5579
	Rec	0.5360	0.5551	0.5748	0.5210	0.6670	0.7931	0.5565
	Gen	0.5365	0.5549	0.5731	0.5214	0.6655	0.7887	0.5559

For each encoding and data set, the maximum (max) and average (avg) AUC was calculated over all algorithms and *p*-value thresholds.

### Classifiers

**Fig 3:**
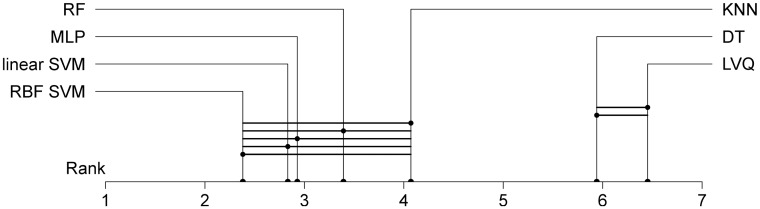
Comparison of classification algorithms. The seven classification algorithms compared by their rank distance over all disease data sets using the additive encoding. A connecting line between encodings means that the null hypothesis of them being significantly different could not be rejected (with *α* = 0.001).

For the comparison of the different classification algorithms, we limited the evaluation of the results to the additive encoding, since it showed the best performance. We also chose to not evaluate the other two encodings separately, since each additional test would increase the risk of getting significant results by chance, increasing the need for multiple test correction. After applying the Friedman test (*k* = 7, *n* = 42) we could reject the null hypothesis of equal performance of the algorithms with (*p* < 10^−15^, see Table 8). In the critical distance diagram (Fig 3) it can be seen that the algorithms form two groups that differ between each other, but no differences can be found within each group (see Table 9 for *p*-values). The better group consists of both SVMs, the Multilayer Perceptron, the Random Forest and the k-nearest neighbor algorithm. The other group is far off to the worse end of the ranking and includes the Decision Tree and the Learning Vector Quantization.

**Table 8:** Average ranks of the seven classification algorithms.

	RBF	Lin	MLP	RF	KNN	DT	LVQ
all	2.38	2.83	2.93	3.39	4.07	5.94	6.45
BD	3.25	3.42	2.58	3.00	3.75	5.50	6.50
CAD	2.17	3.17	2.83	3.83	4.00	5.50	6.50
CD	2.83	3.17	2.83	3.17	3.00	6.50	6.50
HT	2.58	1.92	3.42	4.08	4.42	6.08	5.50
RA	2.50	2.83	2.50	3.50	3.67	6.50	6.50
T1D	1.50	3.17	3.00	3.00	5.00	5.33	7.00
T2D	1.83	2.17	3.33	3.17	4.67	6.17	6.67

The average ranks of the Friedman test for the seven different classifiers using the additive encoding. (Small values are better.) The result of the Friedman test over all data sets is significant (*p* < 10^−15^ for *k* = 7, *n* = 42). The table also shows the average ranks for each data set separately, but the Friedman test is not applicable here because the number of treatments is bigger than the number of problems (*k* = 7, *n* = 6).

**Table 9:** Rank differences and *p*-values for pair-wise comparison of classification algorithms.

Hypothesis	Rank diff	*p*-value
RBF vs LVQ	4.07	< 10^−15^
Lin vs LVQ	3.62	2.465 ⋅ 10^−13^
RBF vs DT	3.56	6.461 ⋅ 10^−13^
MLP vs LVQ	3.52	1.159 ⋅ 10^−12^
Lin vs DT	3.11	6.542 ⋅ 10^−10^
RF vs LVQ	3.06	1.286 ⋅ 10^−9^
MLP vs DT	3.01	2.501 ⋅ 10^−9^
RF vs DT	2.55	7.156 ⋅ 10^−7^
KNN vs LVQ	2.38	4.841 ⋅ 10^−6^
KNN vs DT	1.87	0.0008079
RBF vs KNN	1.69	0.003693
Lin vs KNN	1.24	0.08629
MLP vs KNN	1.14	0.138
RBF vs RF	1.01	0.2228
RF vs KNN	0.68	1
Lin vs RF	0.56	1
RBF vs MLP	0.55	1
DT vs LVQ	0.51	1
MLP vs RF	0.46	1
RBF vs Lin	0.45	1
Lin vs MLP	0.10	1

For each pair-wise comparison of classification algorithms, the rank difference was calculated as the difference of the average ranks over all data sets. The first algorithm of each hypothesis is the better one (lower rank). *p*-values were corrected using Shaffer’s static method.

We also reported the ranks of the algorithms grouped by the different disease data sets for the sake of completeness. However, since the number of compared algorithms exceeded the number of data sets used (*k* = 7, *n* = 6), applying the Friedman test is not meaningful in this context. A comparison of the average AUCs of the algorithms showed that performance differences varied between the different data sets (see Table 10). In the CD data set, for example, all algorithms of the better group achieved average AUCs between 0.5911 and 0.5944 and the other group 0.5495 and 0.5436, showing very low variance in each group, but high variance between groups. This in contrast to the T1D data set, where the AUCs in the first group ranged from 0.8405 to 0.7904 and the second group from 0.7840 to 0.7020.

**Table 10:** Average AUC for each data set and algorithm over all *p*-value thresholds.

AUC	BD	CAD	CD	HT	RA	T1D	T2D
RBF	0.5464	0.5692	0.5944	0.5292	0.7041	0.8405	0.5728
Lin	0.5453	0.5672	0.5911	0.5283	0.6983	0.8331	0.5693
MLP	0.5449	0.5659	0.5922	0.5263	0.6997	0.8357	0.5673
RF	0.5457	0.5629	0.5925	0.5255	0.6900	0.8360	0.5646
KNN	0.5377	0.5583	0.5921	0.5215	0.6834	0.7904	0.5583
DT	0.5253	0.5447	0.5495	0.5100	0.6173	0.7840	0.5461
LVQ	0.5227	0.5314	0.5436	0.5172	0.6222	0.7020	0.5272
Avg	0.5383	0.5571	0.5793	0.5226	0.6736	0.8031	0.5579

All values are AUCs averaged over all *p*-value thresholds for each data set and algorithm. The last row shows the average AUC for each data set over all *p*-value thresholds and algorithms.

## Discussion

### Shared features between data sets

For the *p*-value thresholds from 10^−6^ to 10^−3^ the percentage of shared features was about 20% − 25% for all data sets except RA and T1D. Those latter two disease data sets contained considerably more SNPs reaching the most stringent threshold of 10^−8^ and thus shared up to 80% of the SNPs between to subsets. They were also the two data sets where the AUCs were highest and varied the most between all algorithms.

But even for large fractions of shared SNPs between data sets, the ranks of the classifiers could highly diverge. For example, in the CAD data set the number of SNPs ranged from an average of 3.4 (for *p* < 10^−8^) to 8.0 (for *p* < 10^−6^), but the ranks of four out of the seven classifiers differed by at least three positions (see S2 Table). This suggests that although the data sets were not independent in terms of which features they contained, they were sufficiently independent when used as input for different algorithms.

### Influence of choice of encoding

All three encodings contain the same information about the SNPs and each of them can be converted to the other ones and back. This means that the ability of a classification algorithm to use information depends on its representation and accounts for all found differences in the predictive performance of a classification algorithm.

#### Comparison per classifier

As noted before, the comparison of the three different encodings reveals a clear advantage of the additive encoding for all investigated data sets and classification algorithms. The predictive performance of the algorithms using the additive encoding was on average better for each algorithm than when using another encoding. In all cases, it was either significantly better than at least one of the other two encodings, or no difference could be observed. In addition to performing better in terms of predictive accuracy, the additive encoding has other advantages. For studies with only two possible alleles per SNP, which are the most common studies at the moment, it needs the least storage space while preserving all information among those three encodings. This is beneficial not only because it requires less storage and working memory, but it also has the lowest computational time. The more features an algorithm has to process, the more calculations need to be performed and the more computation time is needed, unless the additional features allow the learned model to converge faster. Therefore, the additive encoding should be chosen in most cases for the investigated algorithms.

The results for Decision Tree were to be expected, since they evaluate single features using discrete rules and the resulting decision function is highly non-linear. Every learned decision tree for one encoding can be transformed into a decision tree for another encoding with the exact same outcome. Apart from constraints like maximum depth they should be almost insensitive to the specific representation of information as long as it is not too complex. Random Forests are ensemble classifiers consisting of a special form of Decision Trees, so they should behave similarly.

It is interesting that the only linear classifier we investigated, the standard linear SVM, showed one of the highest preferences for additive encoding. As shown in [31], the additive encoding can negatively affect the power to detect interactions, especially for linear classifiers. Our results strongly suggest that the number of such risk factors is negligible compared to the number of SNPs with simple additive effects on the phenotype. It could also be possible that there are more complex interactions between SNPs that this study did not capture, but those could only be revealed by much larger cohorts or the investigation of familial cases [49, 50].

#### Comparison per data set

The disease-specific results indicate a relationship between the actual predictive performance and the differences between encodings. The three data sets for which the best predictive performance could be achieved are also the ones for which a significant difference between encodings was observed. The T1D data set has the highest predictive performance of all seven data sets, with some algorithms reaching an AUC of up to 0.87. It also shows the most significant differences between the three encodings with the Friedman test reporting the best *p*-value of < 10^−15^. Next best are RA (AUC up to 0.73, *p* = 2.485 ⋅ 10^−9^) and CD (AUC up to 0.62, *p* = 6.029 ⋅ 10^−8^). For the other diseases no significant effect of the encoding could be observed. This may be due to the small performance differences, which can be masked by random noise more easily the closer the predictions are to random chance. However, upon closer inspection it can be seen that even in the remaining four data sets AUCs of up to 0.58 are reached, so this can not be the only reason. On the other hand, another interesting observation can be made when we look at the results of the five better algorithms on the T1D data set: In each case the additive encoding ranked best, the recessive/dominant encoding second and the genotypic encoding third, without a single exception.

Given that the additive encoding performed the best for all data sets, we can assume that an additive risk model is plausible for most of the SNPs or at least that the impact of additive risk factors outweighs the others. Also, complex SNP-SNP interactions are harder to detect for the machine learning algorithms, especially for small sample sizes.

### Two groups of classifiers

While the question of which encoding to prefer can be answered with satisfying justification, the answer to which of the investigated classification algorithms is best for creating predictive models on SNP data is a bit more ambiguous. After having established the choice of the encoding, the data on which the classifiers are compared was constrained to only a third of the initial performance evaluations. The *p*-value reported by the Friedman test is still highly significant (*p* < 1 ⋅ 10^−13^), but Shaffer’s static method could only separate the classifiers into two groups. Based on our experiments, the linear SVM, the SVM with RBF kernel, the MLP, the Random Forest and the k-nearest neighbor algorithm can not be considered to have statistically significant differences in their predictive performance for these data sets. This does not mean that there is no difference, just that we could not find enough evidence for that claim.

Of course, the predictive performance is not the only criterion after which the classifier should be selected. All algorithms used in this study were implemented in Java and included in the Weka machine learning framework or a wrapper was used to make them accessible for Weka. For each of the algorithms there exist multiple implementations in various languages. It is therefore of only limited usefulness to compare the algorithms by their runtime or their memory efficiency, since it would only be valid for this specific implementation.

More importantly, as already described in Machine Learning Algorithms, the algorithms differ also in how the learned models can be interpreted. If the sole purpose of the classifier is to predict the individual disease risk, this is of no concern. The same holds for the time needed to train the model, since this has to be done only once and it can then be used indefinitely for the usually much faster prediction. But if one wants to gain knowledge about the disease by analyzing how the disease risk is calculated, simple models should be preferred.

From the five algorithms in the better group, the linear SVM can be considered the simplest. The learned model is a linear decision function that is a weighted sum of all input features. The weight for each feature directly correlates with the influence this feature has on the prediction and can be easily extracted. Additionally, there is only one parameter that needs to be fine-tuned during training, making the parameter optimization less time-consuming than other algorithms like, for example, the SVM with RBF kernel or the MLP.

## Conclusions

One of the original ideas behind this study was to investigate whether the disease risk prediction for certain diseases depends on the encoding of the genetic data or not. Each encoding implies a different genetic risk model of how the presence or absence of alleles and genotypes affects the disease risk. It would be naive to assume that all SNPs that are causative for or correlated with the phenotype follow the same risk model. However, the fact that the additive encoding performs best by a large margin indicates that it either corresponds to the underlying genetic risk model, or that it is at least a suitable representation for machine learning, even if the actual risk model is not additive.

While a single “best” algorithm for this kind of data would have been a more favorable outcome, our results suggest that the predictive performance differences between SVM, MLP, RF and even the KNN algorithm are negligible. Further analyses of more and larger data sets may be able to raise the observed performance differences up to a statistically significant level. Researchers choosing one of these popular classification algorithms face a low risk of a substantially worse predictive model. Instead, they can base their decision upon other criteria such as experience with or availability of certain software.

We recommend using the linear SVM, because the learned model directly reflects the influence of individual SNPs on it. If one is only interested in prediction, an SVM with RBF kernel, the Multilayer Perceptron or Random Forest algorithms can be used and offer more possibilities of fine-tuning to individual circumstances.

## Supporting Information

S1 Table AUCs and ranks for the three encodings for all settings. For each data set, *p*-value threshold and classifier, the predictive performances of the risk models of all folds of the cross-validation have been averaged for each encoding scheme and rounded to four decimal places. The corresponding ranks (1 is best) are shown to the right. The settings in each line only differ in the used feature encoding.
(XLS)

S2 Table AUCs and ranks of the seven classifiers for all settings using additive encoding. For each data set and *p*-value threshold, the predictive performances of the risk models of all folds of the cross-validation have been averaged for each classifier and rounded to four decimal places. The corresponding ranks (1 is best) are shown to the right. The settings in each line only differ in the used classifier. (The values in this table are the same as the ‘Additive’ column from S1 Table, but grouped differently for easier comparison.)
(XLS)
